# Imidazolium 3-nitro­benzoate

**DOI:** 10.1107/S1600536809013142

**Published:** 2009-04-18

**Authors:** Guang-Yang Hou, Li-Na Zhou, Qiu-Xiang Yin, Wei-Yi Su, Hui-Lin Mao

**Affiliations:** a School of Chemical Engineering and Technology, Tianjin University, Tianjin 300072, People’s Republic of China

## Abstract

In the title compound, C_3_H_5_N_2_^+^·C_7_H_4_NO_4_^−^, the benzene ring forms a dihedral angle of 40.60 (5)° with the imidizolium ring. The nitro­benzoate anion is approximately planar: the benzene ring makes dihedral angles of 3.8 (3) and 3.2 (1)° with the nitro and carboxyl­ate groups, respectively. In the crystal structure, the cations and anions are linked by inter­molecular N—H⋯O hydrogen bonds, forming a zigzag chain along the *b* axis.

## Related literature

For general background to the physical and biological properties of imidazoles, see: Bunnage & Owen (2008 ▶); Ganellin & Fkyerat (1996 ▶); Weinreb (2007 ▶). For related structures of salts of imidazole with carboxylic acid derivatives, see: Mcdonald & Dorrestein (2001 ▶).
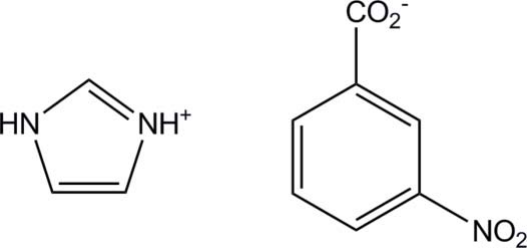


## Experimental

### 

#### Crystal data

C_3_H_5_N_2_^+^·C_7_H_4_NO_4_^−^
*M* *_r_* = 235.20
Monoclinic, 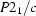

*a* = 12.209 (2) Å
*b* = 12.081 (2) Å
*c* = 7.3216 (15) Å
β = 106.38 (3)°
*V* = 1036.1 (3) Å^3^
*Z* = 4
Mo *K*α radiation
μ = 0.12 mm^−1^
*T* = 298 K
0.38 × 0.21 × 0.13 mm

#### Data collection

Rigaku R-AXIS RAPID IP area-detector diffractometer
Absorption correction: multi-scan (**ABSCOR**; Higashi, 1995 ▶) *T*_min_ = 0.956, *T*_max_ = 0.984
10057 measured reflections
2369 independent reflections
1571 reflections with *I* > 2σ(*I*)
*R*_int_ = 0.051

#### Refinement

*R*[*F* ^2^ > 2σ(*F* ^2^)] = 0.042
*wR*(*F* ^2^) = 0.108
*S* = 1.02
2369 reflections
163 parameters
H atoms treated by a mixture of independent and constrained refinement
Δρ_max_ = 0.20 e Å^−3^
Δρ_min_ = −0.17 e Å^−3^

### 

Data collection: *RAPID-AUTO* (Rigaku/MSC, 2004 ▶); cell refinement: *RAPID-AUTO*; data reduction: *CrystalStructure* (Rigaku/MSC, 2004 ▶); program(s) used to solve structure: *SHELXS97* (Sheldrick, 2008 ▶); program(s) used to refine structure: *SHELXL97* (Sheldrick, 2008 ▶); molecular graphics: *SHELXTL* (Sheldrick, 2008 ▶); software used to prepare material for publication: *SHELXL97*.

## Supplementary Material

Crystal structure: contains datablocks global, I. DOI: 10.1107/S1600536809013142/is2403sup1.cif

Structure factors: contains datablocks I. DOI: 10.1107/S1600536809013142/is2403Isup2.hkl

Additional supplementary materials:  crystallographic information; 3D view; checkCIF report

## Figures and Tables

**Table 1:** Hydrogen-bond geometry (Å, °)

*D*—H⋯*A*	*D*—H	H⋯*A*	*D*⋯*A*	*D*—H⋯*A*
N3—H1⋯O2	0.99 (2)	1.66 (2)	2.6502 (18)	177 (2)
N2—H2⋯O1^i^	0.94 (2)	1.74 (2)	2.677 (2)	175 (2)

Symmetry code: (i) 
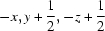
.

**Fig. 1.**
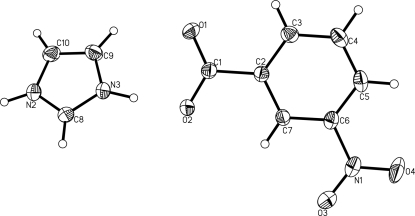
The molecular structure of (I), showing the atom-labelling scheme. Displacement ellipsoids are drawn at the 30% probability level.

**Fig. 2.**
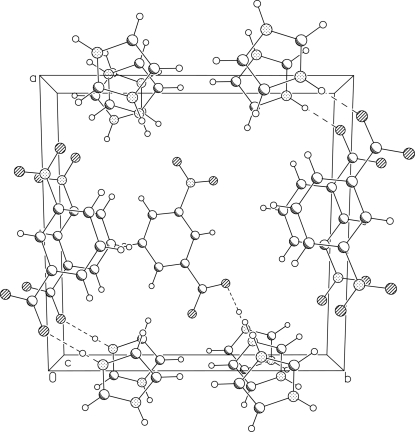
A packing diagram of (I). Dashed lines show N—H···O hydrogen bonds.

## supplementary crystallographic information

### Comment

Imidazole is a commonly utilized substructure within the pharmaceutical industry, as the imidazole ring impart unique physical and biological properties to compounds of interest (Weinreb, 2007; Bunnage & Owen, 2008; Ganellin & Fkyerat, 1996). Synthetic imidazoles are always present in many fungicides and antifungal antiprotozoal, and antihypertensive medications. The crystal structures of salts between nitrobenzoic acids and imidazoles have been analyzed (Mcdonald & Dorrestein, 2001). As an extension study of hydrogen bonding pattern of nitrobenzoic acids and imidazoles herein we report the crystal structure of the title compound, (I).

The structure of the crystal is shown in Fig. 1. The asymmetric unit of the title compund contains one 3-nitrobenzote anion and one imidazolium cation. A proton transfer from the carboxyl group of 3-nitrobenzoic acid to atom N3 of imidazole. The corresponding C8—N3—C9 angle of the imidazole ring is 108.11 (15)°. The dihedral angle between the benzene ring of 3-nitrobenzote and imidazole ring is 40.60 (5)°. And the dihedral angles of the benzene with the nitro and carboxyl groups are 3.8 (3) and 3.2 (1)°, respectively. In the crystal structure, the crystal packing is consolidated by N—H···O intermolecular hydrogen bond.

### Experimental

3-Nitrobenzoic acid and imidazole were mixed in water in a 1:1 molar ratio, then the suspension was heated to 343 K. The clear colourless solution obtained was cooled naturally to room temperature. Colourless crystals were obtained. Then the product was taken out from the solvent by tweezers, and dried in the air at room temperature.

### Refinement

N-bound H atoms were located in a difference Fourier map and refined freely. Other H atoms are placed in calculated positions (C–H = 0.93 Å) and constrained to ride on their parent atoms, with *U*_iso_(H) = 1.2*U*_eq_(C).

### Crystal data

**Table:**

C_3_H_5_N_2_^+^·C_7_H_4_NO_4_^−^	*F*(000) = 488
*M**_r_* = 235.20	*D*_x_ = 1.508 Mg m^−^^3^
Monoclinic, *P*2_1_/*c*	Mo *K*α radiation, λ = 0.71073 Å
Hall symbol: -P 2ybc	Cell parameters from 6213 reflections
*a* = 12.209 (2) Å	θ = 3.4–27.5°
*b* = 12.081 (2) Å	µ = 0.12 mm^−^^1^
*c* = 7.3216 (15) Å	*T* = 298 K
β = 106.38 (3)°	Block, colourless
*V* = 1036.1 (3) Å^3^	0.38 × 0.21 × 0.13 mm
*Z* = 4	

### Data collection

**Table:**

Rigaku R-AXIS RAPID IP area-detector diffractometer	2369 independent reflections
Radiation source: rotating anode	1571 reflections with *I* > 2σ(*I*)
graphite	*R*_int_ = 0.051
ω scans	θ_max_ = 27.5°, θ_min_ = 3.4°
Absorption correction: multi-scan (*ABSCOR*; Higashi, 1995)	*h* = −15→14
*T*_min_ = 0.956, *T*_max_ = 0.984	*k* = −15→15
10057 measured reflections	*l* = −9→9

### Refinement

**Table:**

Refinement on *F*^2^	Secondary atom site location: difference Fourier map
Least-squares matrix: full	Hydrogen site location: inferred from neighbouring sites
*R*[*F*^2^ > 2σ(*F*^2^)] = 0.042	H atoms treated by a mixture of independent and constrained refinement
*wR*(*F*^2^) = 0.108	*w* = 1/[σ^2^(*F*_o_^2^) + (0.0443*P*)^2^ + 0.1735*P*] where *P* = (*F*_o_^2^ + 2*F*_c_^2^)/3
*S* = 1.02	(Δ/σ)_max_ < 0.001
2369 reflections	Δρ_max_ = 0.20 e Å^−^^3^
163 parameters	Δρ_min_ = −0.17 e Å^−^^3^
0 restraints	Extinction correction: *SHELXL97* (Sheldrick, 2008), Fc^*^=kFc[1+0.001xFc^2^λ^3^/sin(2θ)]^-1/4^
Primary atom site location: structure-invariant direct methods	Extinction coefficient: 0.019 (3)

### Special details

**Table:**

Geometry. All e.s.d.'s (except the e.s.d. in the dihedral angle between two l.s. planes) are estimated using the full covariance matrix. The cell e.s.d.'s are taken into account individually in the estimation of e.s.d.'s in distances, angles and torsion angles; correlations between e.s.d.'s in cell parameters are only used when they are defined by crystal symmetry. An approximate (isotropic) treatment of cell e.s.d.'s is used for estimating e.s.d.'s involving l.s. planes.
Refinement. Refinement of *F*^2^ against ALL reflections. The weighted *R*-factor *wR* and goodness of fit *S* are based on *F*^2^, conventional *R*-factors *R* are based on *F*, with *F* set to zero for negative *F*^2^. The threshold expression of *F*^2^ > σ(*F*^2^) is used only for calculating *R*-factors(gt) *etc*. and is not relevant to the choice of reflections for refinement. *R*-factors based on *F*^2^ are statistically about twice as large as those based on *F*, and *R*- factors based on ALL data will be even larger.

### Fractional atomic coordinates and isotropic or equivalent isotropic displacement parameters (Å^2^)

**Table:**

	*x*	*y*	*z*	*U*_iso_*/*U*_eq_	
C1	0.25763 (13)	0.52628 (15)	0.1825 (3)	0.0382 (4)	
C2	0.35926 (13)	0.45955 (14)	0.1673 (2)	0.0337 (4)	
C3	0.34637 (15)	0.36098 (15)	0.0674 (3)	0.0409 (4)	
H3A	0.2733	0.3348	0.0082	0.049*	
C4	0.44008 (17)	0.30055 (16)	0.0538 (3)	0.0470 (5)	
H4A	0.4294	0.2345	−0.0142	0.056*	
C5	0.54890 (16)	0.33750 (16)	0.1401 (3)	0.0463 (5)	
H5A	0.6125	0.2971	0.1332	0.056*	
C6	0.56048 (14)	0.43670 (15)	0.2374 (2)	0.0373 (4)	
C7	0.46907 (13)	0.49843 (14)	0.2524 (2)	0.0352 (4)	
H7A	0.4804	0.5652	0.3183	0.042*	
C8	0.05956 (14)	0.80035 (16)	0.3479 (3)	0.0417 (4)	
H8	0.1048	0.8624	0.3490	0.050*	
C9	0.00096 (16)	0.63117 (17)	0.3339 (3)	0.0509 (5)	
H9	−0.0009	0.5545	0.3227	0.061*	
C10	−0.08242 (16)	0.69552 (17)	0.3607 (3)	0.0485 (5)	
H10	−0.1528	0.6721	0.3719	0.058*	
N1	0.67578 (12)	0.48134 (15)	0.3229 (2)	0.0460 (4)	
H1	0.160 (2)	0.670 (2)	0.304 (3)	0.084 (8)*	
H2	−0.0847 (18)	0.865 (2)	0.384 (3)	0.066 (6)*	
N2	−0.04454 (13)	0.80152 (14)	0.3686 (2)	0.0432 (4)	
N3	0.08885 (12)	0.69827 (13)	0.3259 (2)	0.0423 (4)	
O1	0.16009 (9)	0.48714 (11)	0.1101 (2)	0.0507 (4)	
O2	0.27753 (10)	0.61707 (11)	0.2665 (2)	0.0510 (4)	
O3	0.68555 (10)	0.56865 (13)	0.4101 (2)	0.0574 (4)	
O4	0.75732 (11)	0.42975 (14)	0.3012 (2)	0.0721 (5)	

### Atomic displacement parameters (Å^2^)

**Table:**

	*U*^11^	*U*^22^	*U*^33^	*U*^12^	*U*^13^	*U*^23^
C1	0.0339 (8)	0.0330 (10)	0.0495 (11)	−0.0009 (7)	0.0148 (8)	0.0051 (8)
C2	0.0350 (8)	0.0296 (9)	0.0380 (9)	0.0006 (7)	0.0129 (7)	0.0043 (7)
C3	0.0442 (9)	0.0352 (10)	0.0444 (10)	−0.0038 (8)	0.0143 (8)	0.0009 (8)
C4	0.0624 (12)	0.0311 (10)	0.0527 (11)	0.0026 (8)	0.0246 (10)	−0.0023 (9)
C5	0.0506 (11)	0.0402 (11)	0.0548 (12)	0.0155 (8)	0.0256 (9)	0.0076 (9)
C6	0.0340 (8)	0.0392 (10)	0.0406 (10)	0.0064 (7)	0.0139 (7)	0.0082 (8)
C7	0.0363 (8)	0.0310 (9)	0.0411 (9)	0.0023 (7)	0.0157 (8)	0.0010 (7)
N1	0.0340 (8)	0.0561 (11)	0.0499 (10)	0.0087 (7)	0.0152 (7)	0.0108 (8)
O1	0.0309 (6)	0.0412 (8)	0.0769 (10)	−0.0044 (5)	0.0100 (6)	−0.0008 (7)
O2	0.0354 (6)	0.0370 (8)	0.0836 (10)	−0.0003 (5)	0.0215 (7)	−0.0128 (7)
O3	0.0398 (7)	0.0578 (10)	0.0742 (10)	−0.0063 (6)	0.0154 (7)	−0.0032 (8)
O4	0.0378 (7)	0.0952 (14)	0.0870 (12)	0.0218 (8)	0.0234 (8)	−0.0012 (10)
C8	0.0346 (9)	0.0400 (11)	0.0514 (11)	−0.0031 (7)	0.0137 (8)	−0.0015 (9)
C9	0.0518 (11)	0.0373 (11)	0.0667 (13)	−0.0075 (9)	0.0221 (10)	−0.0067 (10)
C10	0.0375 (9)	0.0534 (13)	0.0583 (12)	−0.0082 (8)	0.0194 (9)	−0.0060 (10)
N2	0.0373 (8)	0.0435 (10)	0.0500 (9)	0.0054 (7)	0.0144 (7)	−0.0031 (7)
N3	0.0352 (8)	0.0413 (9)	0.0523 (9)	0.0039 (7)	0.0153 (7)	−0.0048 (7)

### Geometric parameters (Å, °)

**Table:**

C8—N3	1.307 (2)	C2—C3	1.383 (2)
C8—N2	1.322 (2)	C2—C7	1.391 (2)
C8—H8	0.9300	C3—C4	1.384 (3)
C9—C10	1.339 (3)	C3—H3A	0.9300
C9—N3	1.359 (2)	C4—C5	1.375 (3)
C9—H9	0.9300	C4—H4A	0.9300
C10—N2	1.357 (2)	C5—C6	1.381 (2)
C10—H10	0.9300	C5—H5A	0.9300
N2—H2	0.94 (2)	C6—C7	1.372 (2)
N3—H1	0.99 (2)	C6—N1	1.472 (2)
C1—O2	1.247 (2)	C7—H7A	0.9300
C1—O1	1.252 (2)	N1—O3	1.221 (2)
C1—C2	1.510 (2)	N1—O4	1.2223 (19)
			
N3—C8—N2	109.25 (16)	C7—C2—C1	119.68 (15)
N3—C8—H8	125.4	C2—C3—C4	121.27 (17)
N2—C8—H8	125.4	C2—C3—H3A	119.4
C10—C9—N3	107.59 (17)	C4—C3—H3A	119.4
C10—C9—H9	126.2	C5—C4—C3	120.45 (18)
N3—C9—H9	126.2	C5—C4—H4A	119.8
C9—C10—N2	106.85 (16)	C3—C4—H4A	119.8
C9—C10—H10	126.6	C4—C5—C6	117.64 (16)
N2—C10—H10	126.6	C4—C5—H5A	121.2
C8—N2—C10	108.20 (16)	C6—C5—H5A	121.2
C8—N2—H2	125.1 (14)	C7—C6—C5	123.07 (17)
C10—N2—H2	126.7 (14)	C7—C6—N1	117.95 (16)
C8—N3—C9	108.11 (15)	C5—C6—N1	118.93 (15)
C8—N3—H1	128.8 (14)	C6—C7—C2	118.93 (16)
C9—N3—H1	123.1 (14)	C6—C7—H7A	120.5
O2—C1—O1	124.82 (16)	C2—C7—H7A	120.5
O2—C1—C2	117.12 (14)	O3—N1—O4	123.05 (17)
O1—C1—C2	118.06 (16)	O3—N1—C6	118.65 (14)
C3—C2—C7	118.62 (15)	O4—N1—C6	118.29 (17)
C3—C2—C1	121.69 (15)		
			
N3—C9—C10—N2	0.1 (2)	C3—C4—C5—C6	−0.8 (3)
N3—C8—N2—C10	0.5 (2)	C4—C5—C6—C7	0.5 (3)
C9—C10—N2—C8	−0.4 (2)	C4—C5—C6—N1	−176.88 (16)
N2—C8—N3—C9	−0.4 (2)	C5—C6—C7—C2	0.5 (3)
C10—C9—N3—C8	0.2 (2)	N1—C6—C7—C2	177.96 (14)
O2—C1—C2—C3	−176.11 (16)	C3—C2—C7—C6	−1.3 (2)
O1—C1—C2—C3	3.7 (3)	C1—C2—C7—C6	179.92 (15)
O2—C1—C2—C7	2.6 (2)	C7—C6—N1—O3	3.0 (2)
O1—C1—C2—C7	−177.59 (16)	C5—C6—N1—O3	−179.45 (16)
C7—C2—C3—C4	1.0 (3)	C7—C6—N1—O4	−176.07 (16)
C1—C2—C3—C4	179.79 (16)	C5—C6—N1—O4	1.5 (2)
C2—C3—C4—C5	0.0 (3)		

### Hydrogen-bond geometry (Å, °)

**Table:**

*D*—H···*A*	*D*—H	H···*A*	*D*···*A*	*D*—H···*A*
N3—H1···O2	0.99 (2)	1.66 (2)	2.6502 (18)	177 (2)
N2—H2···O1^i^	0.94 (2)	1.74 (2)	2.677 (2)	175 (2)

Symmetry codes: (i) −*x*, *y*+1/2, −*z*+1/2.
